# CanMEDS and the discourse of nature: on petals, beauty and the symmetry of flowers in the training of physicians

**Published:** 2012-09-30

**Authors:** Jonathan White, Shannon Erichsen

**Affiliations:** Department of Surgery, University of Alberta, Edmonton, Alberta, Canada

It is with much interest that we read recent exchanges which employed the discourses of threat and protection to explore the genesis and nature of the CanMEDs framework.^1^,^2^ Different discourses allow us to consider familiar subjects from a new perspective, and it is thus that we would like to suggest an alternative discourse which has arisen in our day-to-day use of CanMEDs: namely the discourse relating to nature.

This discourse includes objects such as flowers and petals, concepts such as beauty, growth, natural variation and roles such as gardeners and that which grows. We believe that this discourse has merit as a point of view, and may also have an important application when discussing CanMEDs with medical trainees.

The choice of the familiar flower-shaped construct consisting of petals overlapping around a central point suggests that those who created CanMEDs may have been aware of similar shapes existing in nature, that they considered such shapes beautiful, and that they might have believed that the appropriate arrangement of the various competencies of a doctor possessed some degree of natural beauty. The choice of a flower further suggests simplicity, innocence and purity, which could be viewed as implying perhaps that the physician described by CanMEDs is perfect, pure or ideal, or in some way unassailable, and also that the arrangement of roles in the framework is perhaps natural, organic or innate.

The central location for ‘Medical Expert’ in the flower suggests that it may be ‘at the heart’ of each doctor’s professional identity. Assuming that those who created CanMEDs had at least a passing acquaintance with botany, the radial arrangement of the petals may be interpreted to mean that each of the roles originates within, grows out from and remains attached to the Medical Expert at the centre. Absence or deficiency of one or more petals would naturally detract from the natural beauty of the whole flower.

It is common to observe flowers that lack perfect symmetry and that demonstrate inequality of petal size. To illustrate the importance of this point for the application of the CANMeds roles to medical education, we provide the following recent conversation between a medical trainee (T) and one of the authors (J):

T: Look, I don’t see the point of this CanMEDs thing.

J: Um, well, it’s a Royal College framework, right?

T: I know, I know, but I just don’t buy it. I mean, I get Medical Expert, but what about all the other parts – what are they for?

J: They’re trying to tell you that there are lots of things you have to be good at to be a great doctor. Look, think about it like this: CanMEDs is a flower, a beautiful flower. When you graduate, you’ll be a Medical Expert. That’s at the middle of the flower, and you can’t be a doctor without that. But don’t forget about all the petals. You don’t have much of a flower without petals, right? You need to work to make sure you’ve got something on each of the petals. Ok, so you’re a good Communicator, or a good Collaborator. But don’t forget about being an Advocate for your patient or being a good Manager when you’re in charge. I’m not suggesting all of your petals have to be exactly the same size when you leave residency, but you have to have something in each of them. Maybe your Professional is a nice-sized petal but your Scholar needs to grow a little – you can work on that. We provide the water to help you grow in residency, but it’s your job to make sure you’re not missing any petals. We want all of you to be complete CanMEDs flowers, even if you’re all slightly different. No-one wants to grow up to be a misshapen flower with missing petals.

The production of beautiful flowers requires careful planning, appropriate selection of soil and seed, frequent watering, and dutiful nurture. We believe that the discourses of nature and horticulture may also provide us with a way to consider aspects of the training of physicians. It is interesting to consider which seeds might grow best in which soils (trainees and programs), how frequently the flower should be watered (teaching) and inspected (assessment) and how much sun and rain (positive and negative experiences) might be required for optimal growth. These discourses also allow us to consider more interesting philosophical questions. Is the process of growth passive, or does the seed play an active role? Who is responsible when a seed does not germinate? And which is most essential to the quality of the flower produced: the good seed or the expert gardener?

In summary, we believe that a range of discourses are required to help us think about the training of physicians. Our flower metaphor grounded in a discourse of nature allows us to see many positive and intriguing aspects of the CanMEDs roles for both trainees and teachers. We hope our readers have found our exploration helpful and encourage others to suggest additional interesting and valuable discourses.
